# SMND-309 activates Nrf2 signaling to alleviate acetaminophen-induced hepatotoxicity and oxidative stress

**DOI:** 10.1371/journal.pone.0310879

**Published:** 2025-03-31

**Authors:** Yao Dong, Ru Jia, Yujie Jiang, Qing Li, Lei Wang, Wensi Ding, Rui Yan, Yujie Qiu, Zhengjie Shi, Wenying Liu, Jing Wang, Sen Xu, Na Li

**Affiliations:** 1 Binzhou Medical University, Yantai, Shandong, P.R.China; 2 Department of Obstetrics, Yantai Affiliated Hospital of Binzhou Medical University, Yantai, Shandong, P.R.China; 3 Department of Gastroenterology, Yantai Zhifu Hospital, Yantai, Shandong, P.R.China; 4 Department of Orthopedics, Yantai Yantaishan Hospital, Yantai, Shandong, P.R.China; Jadavpur University, INDIA

## Abstract

**Background:**

Acetaminophen (APAP) can be used for pain relief and fever alleviation, the overdose of which, however, may lead to the accumulation of N-acetyl-p-benzoquinone imine (NAPQI), inducing oxidative stress and liver damage. The natural compound SMND-309 has been shown to have hepatoprotective effects and potential antioxidant activity. However, its ability to alleviate acetaminophen-induced acute liver injury (AILI) has not been elucidated.

**Objective:**

To explore the protective effect of the natural compound SMND-309 against AILI and the potential mechanism.

**Methods:**

The AILI model was established using a mouse model and HepG2 cells for pathological evaluation and biochemical assays of mouse liver tissues to assess the level of liver injury. The effects of SMND-309 on cellular ROS levels and mitochondrial membrane potential were detected using DCFH-DA and JC-1 probes. Western blotting was performed to detect the expressions of Nrf2 signaling pathway and key proteins related to APAP metabolism in the combination of immunohistochemistry of liver tissues, with immunofluorescence assay used to detect whether Nrf2 undergoes nuclear translocation. Molecular docking, molecular dynamics simulation (MD) and biofilm layer interference (BLI) experiments were performed to detect the interaction of SMND-309 with Keap1.

**Results:**

SMND-309 improved histopathological changes in the liver, decreased alanine aminotransferase (ALT), aspartate aminotransferase (AST), and lactate dehydrogenase (LDH) levels, as well as attenuated oxidative stress injury and mitochondrial dysfunction in the HepG2 cell line. Further studies revealed that SMND-309 promoted nuclear translocation of Nrf2 and upregulated the expressions of glutamate-cysteine ligase catalytic subunit (GCLC), heme oxygenase 1 (HO-1) and NAD(P)H quinone dehydrogenase 1 (NQO1). In addition, molecular docking and MD suggested that SMND-309 could bind Keap1 and identified possible binding modes, with BLI experiments confirming that SMND-309 directly interacted with Keap1.

**Conclusion:**

SMND-309 exerts hepatoprotective effects against AILI in an Nrf2-ARE signaling pathway-dependent manner.

## 1. Introduction

Acetaminophen (APAP) is a commonly used non-steroidal anti-inflammatory drug (NSAID) with good antipyretic and analgesic effects. However, excessive intake of APAP may cause severe liver toxicity, known as APAP-induced liver injury (AILI) [1,2]. In the United States, nearly half of the cases of acute liver injury are associated with APAP [3], with extremely high morbidity and mortality if left untreated [4]. Oxidative stress has been shown to be an important cause of APAP-induced hepatotoxicity [5]. Excess APAP is metabolized by the cytochrome P450 (CYP450) enzyme system to produce the toxic metabolite N-acetyl-p-benzoquinone imine (NAPQI), which rapidly depletes intracellular glutathione (GSH) to trigger oxidative stress and mitochondrial dysfunction, ultimately leading to cell necrosis [6]. There is a lack of specific drugs for AILI. N-acetylcysteine (NAC) is the only FDA-approved drug for the treatment of AILI, which is used to promote GSH synthesis. However, its clinical application is limited by severe side effects and a limited therapeutic time window [7]. Therefore, it is particularly important to find novel drugs and methods for treating AILI.

Salvia miltiorrhiza Bunge is a traditional medicinal plant used in East Asia for the treatment of liver diseases [8,9], with the active ingredient Salvianolic acid B (Sal B) in its rhizomes having a wide range of pharmacological activities such as resistance to cellular oxidative stress, treatment of acute liver injury, and anti-inflammatory effects [10–12]. SMND-309 is the active metabolite of Sal B *in vivo*, the main active ingredient in Salvia miltiorrhiza Bunge, with a variety of pharmacological activities. Studies have shown that SMND-309 exhibits a protective effect against reperfusion injury in neuronal cells [13] and an anti-chronic intermittent hypoxia-induced lung injury [14]. Notably, Hou et al. reported that SMND-309 can alleviate carbon tetrachloride-induced hepatic injury and restore the normal tissue structure of hepatic lobules to a certain extent [15]. In addition, SMND-309 showed stronger pharmacological activity compared with Sal B. However, there was no study about the therapeutic effect of SMND-309 on AILI.

In the therapeutic strategy of AILI, the activation of hepatocyte antioxidant defense system centered on nuclear factor erythroid 2-related factor 2 (Nrf2) is of great significance and application prospect [1]. Studies have shown that some polyphenols, terpenoids, and anthraquinones natural products can activate this system to reduce oxidative stress damage in AILI [16,17]. Nrf2 plays a crucial role in the physiological and pathological process of AILI as a key transcription factor in the anti-oxidative stress signaling pathway. Under normal conditions, the activity of Nrf2 is negatively regulated by the upstream protein Kelch-like ECH-associated protein 1 (Keap1), which interacts with antioxidant response elements (AREs) in the genome to regulate the expression of downstream genes and protect the liver from oxidative stress damage [18]. The study by Chan and Enamoto et al. further demonstrated that Nrf2 knockout mice had a higher susceptibility to APAP than wild-type mice [19,20], which emphasizes the critical role of the Nrf2 signaling pathway in counteracting APAP-induced hepatotoxicity. These studies suggest the potential of the Nrf2-ARE signaling pathway as an effective target against AILI and provide a scientific basis for the development of new therapeutic approaches.

Given that oxidative stress is a key factor in APAP-induced liver injury and that SMND-309 has potential oxidative stress-relieving and hepatoprotective effects, the study aims to investigate whether SMND-309 can alleviate AILI by alleviating oxidative stress and to preliminarily explore its potential mechanism of action.

## 2. _Materials and methods_

### 2.1. Chemicals and reagents

APAP was sourced from Shanghai Aladdin Biochemical Technology Co., Ltd., Shanghai, China (A105807). SMND-309 and ML385 was procured from MedChemExpress, NJ, USA (HY-13056, HY-100523). The BCA analysis kit, reactive oxygen species assay kit, mitochondrial membrane potential assay kit and Nuclear and Cytoplasmic Protein Extraction Kit, along with RIPA Lysis Buffer, were sourced from Solarbio Science & Technology Co., Ltd., Beijing, China. The kits used to measure alanine aminotransferase (ALT), aspartate aminotransferase (AST), lactate dehydrogenase (LDH), glutathione (GSH), malondialdehyde (MDA), Catalase (CAT), total superoxide dismutase (T-SOD), were purchased from Elabscience (Wuhan, China). Antibodies against Nrf2, β-actin, and goat anti-rabbit IgG (H + L) HRP were sourced from Bioworld Technology, Bloomington, MN, USA (BS6286, AP0060, ZJ2020-R). Antibodies against HO-1, NQO1, and GCLC were obtained from Proteintech Group, Inc., Chicago, USA (66743-1-Ig, 67240-1-Ig, 12601-1-AP). Lamin B1 polyclonal antibody was purchased from Abcam, Cambridge, MA, USA (ab16048). Antibodies against Keap1 were obtained from ABclonal Technology Co., Ltd., Wuhan, China (A25951). Streptavidin biosensors (SA) were obtained from Sartorius, Göttingen, Germany. BL21(DE3) E. coli cells, isopropyl-β-d-thiogalactoside, and Kanamycin were sourced from Tsingke Biotechnology Co., Beijing, China, and Solarbio, Beijing, China (TSC-E01, I8070, K8020-1). His-tag protein purification Magbeads were obtained from Absin, Shanghai, China (abs9901). Zeba Spin Desalting Columns 7K MWCO were procured from Thermo Fisher Scientific, Waltham, MA, USA (89882). Sulfo-NHS-LC-biotin was sourced from ApexBio Technology, Houston, TX, USA (A8003).

### 2.2. Biochemical analysis

Mouse blood samples were collected into blood collection tubes and placed in an ice cube box for proper cooling, followed by centrifuging at 10,000 × g for 10 min at 4°C to collect serum. The serum levels of AST, ALT, and LDH were then measured using commercial kits according to the instructions. The levels of GSH, CAT, MDA, and SOD in HepG2 cells and liver tissue were determined using commercial kits according to the manufacturer’s instructions.

### 2.3. _Cell culture_

The HepG2 human hepatocyte cell line was obtained from the Cell Bank of the Chinese Academy of Sciences (Shanghai, China). Cells were cultured at 37°C in 1,640 medium supplemented with 1% penicillin-streptomycin (Gibco, USA) and 10% [v/v] fetal bovine serum (AusGenex, Australia) in a humidified environment containing 5% CO_2_.

### 2.4. Cell viability assay

HepG2 in the logarithmic growth phase was taken to be washed with PBS and centrifuged (1500r, 3min), followed by diluting by adding serum-containing medium to achieve a cell density of 2 × 10^5^ cells/mL after removing the supernatant. 96-well culture plates were used to spread the plates, with 100 μL of well-mixed cell suspension added to each well and cultured overnight in a 5% CO^2^ incubator. Subsequently, serum-free medium containing different concentrations of SMND-309 (0, 2.5, 5, 10, 20,40, 80 μM) was applied to HepG2 cells for 24 h and 48 h to detect the toxicity of SMND-309 to HepG2 cells. To investigate the protective effect of SMND-309 on the cells, the cells were preincubated with SMND-309 (10 and 40 μM) for 24 h, followed by treatment with APAP (10 mM) for 24 h. To evaluate the effect of Nrf2, HepG2 was pretreated with ML385 (10 μM) for 24 h, followed by treatment with SMND-309 (40 μM, for 24 h) and 10 mM APAP for 24 h. After that, 10 μl of MTT solution was added to each well and incubated for 4 hours, with the formed blue methanesulfonate dissolved in DMSO with gentle shaking and the absorbance was at 490 nm using a microplate reader (Thermo Fisher). Statistical results were standardized to the NC group and expressed using percentages.

### 2.5. Measurement of mitochondrial membrane potential (ΔΨm)

HepG2 was inoculated in 6-well plates for pre-culture using SMND-309 for 24 h, followed by exposure to APAP for continued action for 12 h and the addition of JC-1 staining solution (5 μg/ml) for incubating 20 min at 37°C. Subsequently, the cells were co-incubated with JC-1 staining solution (5 μg/ml) for 20 min at 37°C. In the physiological state, JC-1 aggregates in the mitochondrial matrix to produce red fluorescence. At lower ΔΨm, JC-1 cannot aggregate in the mitochondrial matrix, at which time JC-1 is a monomer (monomer) producing green fluorescence. The mitochondrial membrane potential was analyzed using laser scanning confocal microscopy (Leica, Germany) in the setting of red fluorescence and green fluorescence, with Δψm of HepG2 cells expressed as the ratio of fluorescence intensity of JC-1 aggregates to JC-1 monomer.

### 2.6. Cell modeling and detection of reactive oxygen species

HepG2 cells were grown in six-well plates and incubated with 10, 40 μM SMND-309 for 24 hours. Subsequently, cells were additionally treated with 10 mM APAP for 2 h. Intracellular ROS were detected using the fluorescent probe DCFH-DA as described by Li [21]. Flow cytometry (BD Biosciences, USA) and fluorescence microscopy (Leica, Germany) helped acquire images and quantify ROS levels at the single-cell level.

### 2.7. Western blot analysis

HepG2 cells were lysed using RIPA buffer after digestion, with proteins separated by SDS-PAGE and transferred to a PVDF membrane. After transfer, the membranes were incubated with primary antibodies, followed by secondary antibody incubation combined with horseradish peroxidase. Protein bands were visualized using an ECL detection system (Tanon, China). Band intensities were quantified using ImageJ software, with β-actin or Lamin B1 used as reference proteins.

### 2.8. Animals and ethical statements

Female BALB/c mice at six weeks old weighing approximately 20 ±  2 g were provided by Jinan Pengyue Experimental Animal Breeding Co., Ltd. The mice were kept in a laminar flowing cabinet under specific pathogen-free conditions with a temperature range of 22-26°C and humidity of 45-75% for a 12-hour day/night cycle. Animal experiments were approved by the Animal Research Committee of Binzhou Medical University (License No. 2024-L041) and were conducted according to the guidelines of the National Research Council.

### 2.9. AILI model and SMND-309 treatment

**Fig 1:**
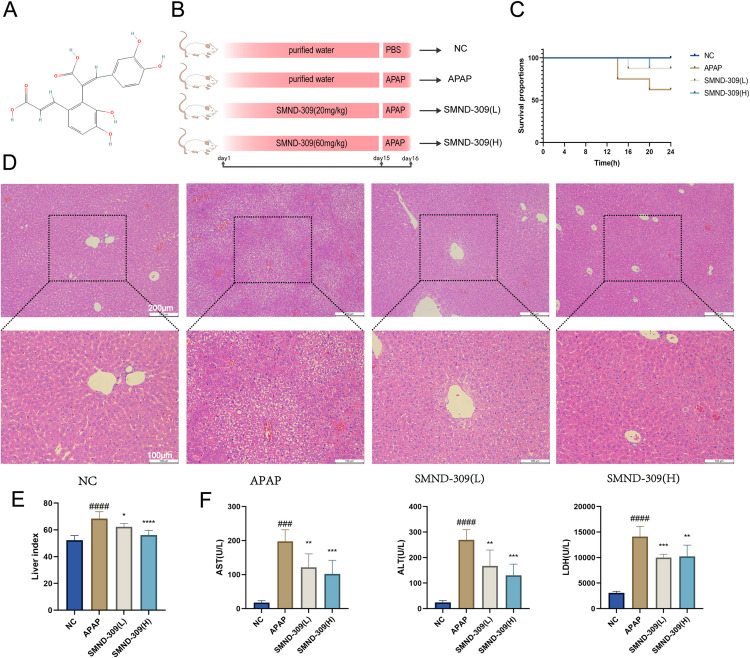
Protective effect of SMND-309 against liver injury in acute liver injury model mice. In the study, mice were orally administered different doses of SMND-309 (20 mg/kg and 60 mg/kg) for 14 consecutive days, followed by the induction of AILI by intraperitoneal injection of 400 mg/kg of APAP. A. Two-dimensional images of the chemical structure of SMND-309. B. The design of the animal experiments was depicted using simulation diagrams, including the setup of the different experimental groups, with NC representing the normal control group, APAP representing the model group given APAP only, SMND-309 (L) representing the treatment group pretreated with a low dose of SMND-309 (20 mg/kg), and SMND-309 (H) representing the treatment group pretreated with a high dose of SMND-309 (60 mg/kg). C. Mice in each group were evaluated by H&E staining for histologic changes in the liver, with the experimental groupings consistent with Fig B. Low and high magnification images were acquired separately under the same field of view. D. Serum levels of aspartate aminotransferase (AST), alanine aminotransferase (ALT), and lactate dehydrogenase (LDH) were measured in each group of mice to assess the degree of liver injury. #p <  0.05, ##p <  0.01, ####p <  0.001, ####p <  0.0001 indicate significant differences compared with the NC group, and * p <  0.05, **p <  0.01, ***p <  0.001, ****p <  0.0001 indicate significant differences compared with the APAP group (Statistical plots of the data are expressed as mean ±  standard deviation).

The experimental setup is depicted in Fig 1B. Thirty-two mice were divided randomly into four groups (n = 8): a normal control (NC) group, an APAP model group, a high-dose SMND-309 group (SMND-309-high, 60mg/kg), and a low-dose SMND-309 group (SMND-309-low, 20mg/kg). Both SMND 309 and APAP were prepared in purified water for oral administration. On the 15th day of the experiment, the NC group received PBS via gavage, while the other groups received an APAP dose of 400 mg/kg through the same method. Mice were euthanized 24 hours after APAP administration via an injection of 0.15 mL of 1% pentobarbital sodium, and liver tissues were immediately collected for analysis. The research protocol received approval from the Medical Ethics Committee of Binzhou Medical University and complied with the National Research Council's Guide for the Care and Use of Laboratory Animals.

### 2.10. Immunofluorescence

Drug-treated HepG2 cells were inoculated on coverslips and cultured at 37°C with 5% CO_2_ until monolayer formation, followed by fixing HepG2 cells using 4% paraformaldehyde (Biosharp). Cell permeabilization was subsequently performed using 0.2% Triton-100 (Biosharp), and non-specific binding sites were closed using PBST containing 1% BSA for 30 min. Cell-attached slides were incubated with rabbit anti-Nrf2 antibody (1:100) overnight at 4°C, followed by incubation with goat anti-rabbit IgG antibody (1:2000) for 1 hour at 37°C, with cell nuclei stained using DAPI. Finally, images were observed and recorded using a fluorescence microscope. All experimental manipulations were performed away from light to prevent fluorescence quenching.

### 2.11. Histopathological evaluation

Liver tissue specimens were preserved in 4% paraformaldehyde solution and embedded in paraffin. After the samples were cut into 5-μm-thick sections, the paraffin sections were dehydrated with the sections sequentially placed in Eco-friendly dewaxing solution Ⅰ for 10 min, Eco-friendly dewaxing solution Ⅱ for 10 min, Eco-friendly dewaxing solution Ⅲ for 10 min, anhydrous ethanol Ⅰ for 5 min, anhydrous ethanol Ⅱ for 5 min, anhydrous ethanol Ⅲ for 5 min, and rinsed with distilled water. The sections were then stained with hematoxylin staining solution (Solarbio) for 5 min and eosin staining solution (Solarbio) for 30 sec, during which time they were rinsed with tap water for 10 min to remove the excess staining solution. Finally, the sections were dehydrated in 75% alcohol for 5 min, 85% alcohol for 5 min, anhydrous ethanol Ⅰ for 5 min, anhydrous ethanol Ⅱ for 5 min, n-butanol for 5 min, xylene Ⅰ for 5 min, with the sections sealed with sealing-section glue. Images were acquired through a microscope (Leica).

### 2.12. Immunohistochemistry

Paraffin sections were first deparaffinized in immunohistochemistry experiments, followed by thermal remediation using EDTA pH 8.0 for 30 min. After repair, the sections were cooled naturally and washed with PBS (pH 7.4) for 5 min each by shaking on a decolorizing shaker 3 times. Then, endogenous peroxidase was blocked and the sections were incubated in 3% hydrogen peroxide solution for 25 min at room temperature away from light, followed by washing with PBS (PH7.4) 3 times for 5 min each time. The sections were closed with 3% BSA for 30 min, then diluted primary antibody was added dropwise to the sections and incubated at 4°C overnight. After washing with PBS, a secondary antibody (HRP-labeled) of the corresponding species to the primary antibody was added dropwise and incubated for 50 min at room temperature. The nuclei were highlighted using DAB staining (Solarbio) along with hematoxylin staining (Solarbio). Finally, the sections were dehydrated and sealed, with images acquired by microscopy (Leica).

### 2.13. Liver index assay

The liver index was calculated based on the following formula: liver index of mice  =  liver weight (mg)/body weight (g).

### 2.14. Quantitative real-time PCR (RT-qPCR)

RNA extraction was performed from HepG2 cells using Trizol reagent (Vazyme) and the concentration of RNA was determined by NanoDrop spectrophotometer (ThermoFisherScientific, USA). RNA was reverse transcribed into cDNA by reverse transcription reagent (Vazyme), with gene expression data normalized by housekeeping gene β-actin. Subsequent data analysis was performed using the 2^-ΔΔCt method. The primers used in the study are listed in Table 1.

**Table 1:** Primer sequences for qPCR.

Gene		Sequence (5’ to 3’)
NQO1	FORWARD REVERSE	TGGCTAGGTATCATTCAACTC CCTTAGGGCAGGTAGATTCAG
GCLC	FORWARD REVERSE	GGCGATGAGGTGGAATAC AAAGGGTAGGATGGTTTGG
HO-1	FORWARD REVERSE	TGCGGTGCAGCTCTTCTG GCAACCCGACAGCATGC
GAPDH	FORWARD REVERSE	CTGCACCACCAACTGCTTAG AGGTCCACCACTGACACGTT

### 2.15. Expression, purification and biotinylation of recombinant Keap1 protein

The recombinant Keap1 construct was cloned into the pET30a vector and transformed into BL21(DE3) E. coli cells. Monoclonal colonies containing kanamycin (25 μg/mL) were identified on LB agar plates. Overnight cultures from single colonies in LB medium were incubated with the addition of kanamycin at 37°C with constant shaking. Next, these cultures were used to inoculate mass cultures at a 1:400 dilution to achieve an optical density (OD600) between 0.4 and 0.6, with protein expression induced by adding 0.1 mM IPTG. Then, the cultures were incubated at 16°C with constant shaking for 16 h allowing continued Keap1 protein expression. After sonication, the cells were added with binding buffer (20 mM sodium phosphate, 500 mM NaCl, 10 mM imidazole) and PSMF to be purified by purification with His-tagged protein purification magnetic beads. All protein samples were desalted using a Zeba rotary desalting column (7K MWCO) according to the manufacturer’s instructions. After purification, protein samples were prepared for subsequent Biolayer Interferometry analysis (BLI analysis, Fortebio Octet 96e, Sartorius, Germany) by biotinylating them using Sulfo-NHS-LC-biotin.

### 2.16. Molecular docking analysis

Molecular docking was performed using Discovery Studio 2019 (Dassault Systèmes, France). The Keap1 crystal structure (PDB:3WN7 [21]) was processed, including the addition of missing atoms, removal of water molecules, and assignment of partial charges. Ligand structures were prepared by generating 3D conformations and assigning partial charges. Molecular docking (LibDock) was performed using default settings, with docking results evaluated to determine the most favorable binding site. Finally, molecular visualization and overlay analysis were performed using PyMOL (Schrödinger Inc., USA).

### 2.17. Molecular dynamics simulations

Molecular dynamics simulations (MD) were performed using the Gromacs2022 program with GAFF force field for small molecules, AMBER14SB force field and TIP3P water model for proteins, merging the files of proteins and low molecular weight ligands to construct the simulation system of the complex, which was carried out at constant temperature and pressure as well as under periodic boundary conditions. During the MD simulations, all involved hydrogen bonds were constrained using the LINCS algorithm with an integration step of 2 fs. Electrostatic interactions were calculated using the PME (Particle-mesh Ewald) method with the cutoff value set to 1.2 nm. The cutoff value for non-bonded interactions was set to 10 Å and updated every 10 steps. The simulation temperature was controlled by the V-rescale temperature coupling method at 298 K, and the pressure was controlled by the Berendsen method at 1 bar. 100 ps NVT and NPT equilibrium simulations were performed and 100 ns MD simulations were performed for the complex system at 298 K, with the conformation saved every 10 ps. After the simulations, the simulation trajectories were analyzed using VMD and Pymol, and the MMPBSA binding free energy analysis between the protein and the low molecular weight ligand was performed using the g_mmpbsa program.

### 2.18. Biofilm Layer Interference Analysis (BLI)

Protein-ligand interactions were evaluated using the Octet RED96e system. After pre-hydration of SA biosensors in PBST for 10 min, biotinylated protein samples were loaded onto these biosensors with a baseline established as a reference. Then, a double subtraction method was implemented to prepare two sets of biosensors: a bound protein biosensor and a protein-free reference biosensor, which were subjected to the same concentration of SMND-309 for a 100-second conjugation phase and a 100-second dissociation phase, respectively. Data acquisition was performed at 25°C in a black 96-well plate shaken at 1,000 rpm. Protein sensor data were subtracted from reference biosensor data to correct for non-specific binding.

### 2.19. Statistical analysis

All data are expressed as the mean ±  standard deviation (Mean ±  SD) of at least three independent experiments per sample. A one-way ANOVA (One-way ANOVA) was performed in experiments comparing three or more sets of data. Statistical analysis and plotting were performed using GraphPad Prism 8.0 software, with a p-value of less than 0.05 considered statistically significant.

## 3. Results and analysis

### 3.1. SMND-309 alleviated acute hepatic injury *in vivo*

To test whether SMND-309 has hepatoprotective drug activity, an animal model was first established to investigate the hepatoprotective potential of SMND-309 in AILI model mice. Fig 1A and Fig 1B demonstrate the chemical structure of SMND-309 and the design of animal experiments. The survival of mice within 24 h after administration of excess APAP for modeling was shown as a survival curve (Fig 1C). First, the histopathological assessment of liver tissues was performed by hematoxylin-eosin (H&E) staining, with the results shown in Fig 1D that the liver tissues of mice in the normal control (NC) group presented an intact hepatic lobular structure, with hepatic cords arranged in a radial pattern, without any necrosis, degeneration, or inflammatory cell infiltration. In contrast, liver tissues in the APAP-treated group showed significant degeneration and inflammatory cell infiltration. SMND-309 (20 and 60 mg/kg) treatment significantly attenuated the degeneration and necrosis of liver tissues, restored the normal organization of the hepatic cords, and reduced inflammatory cell infiltration. Next, serum levels of alanine aminotransferase (ALT), aspartate aminotransferase (AST), and lactate dehydrogenase (LDH) were detected to assess the hepatoprotective effects of SMND-309. As shown in Fig 1E, the liver index of SMND-309-treated mice was normalized, and the serum levels of AST, ALT and LDH were significantly reduced compared with the model group (Fig 1F), suggesting that SMND-309 pretreatment can alleviate acute liver injury in mice.

### 3.2. Effect of SMND-309 on HepG2 cell viability after exposure to excessive APAP

**Fig 2:**
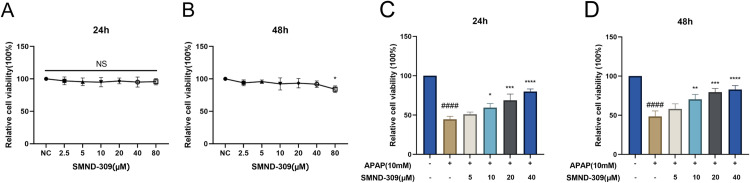
Effect of SMND-309 on cell viability and protection against excess APAP-induced damage in HepG2 cells. HepG2 cells were cultured in 96-well plates and co-incubated with different concentrations of SMND-309 (0-80 μM) for 24 h and 48 h, with the cell viability measured by MTT assay. A. Effect of SMND-309 on the viability of HepG2 cells at 24 h. B. Effect of SMND-309 on the viability of HepG2 cells at 48 h. C. Protective effect of SMND-309 pretreatment for 24 h against excess APAP-induced cell damage. D. Protective effect of SMND-309 pretreatment for 48 h against excess APAP-induced cell damage. (Statistical plots of the data are expressed as mean ±  standard deviation, with sample size **n** =  3).

Before the cellular experiments, the potential toxicity of SMND-309 on HepG2 cells was first evaluated by MTT assay, with the experimental results showing that SMND-309 did not significantly affect the viability of HepG2 cells in the concentration range of 0-80 μM during the 24 h exposure time (Fig 2A). However, SMND-309 began to exert an inhibitory effect on cell viability when the incubation time was extended to 48 hours, especially at the high concentration of 80 μM (p < 0.05), without any significant effect on HepG2 cell viability in the concentration range of 0-40 μM (Fig 2B). Furthermore, 10 mM [22] APAP was selected to establish an *in vitro* model to explore the cytoprotective potential of SMND-309. After 24 h of APAP treatment, the viability of HepG2 cells was significantly decreased (p <  0.0001) (Fig 2C-D). It was found that the pretreatment of cells with SMND-309 for 24h and 48h can alleviate APAP-induced cytotoxicity to a certain extent, with the protective effect beginning to become significant especially at concentrations above 10 μM (p <  0.05), with the most pronounced effect of SMND-309 on enhancing cell viability at a concentration of 40 μM.

### 3.3. Effects of SMND-309 on APAP induced oxidative stress and mitochondrial dysfunction *in vitro* and *in vivo*

**Fig 3:**
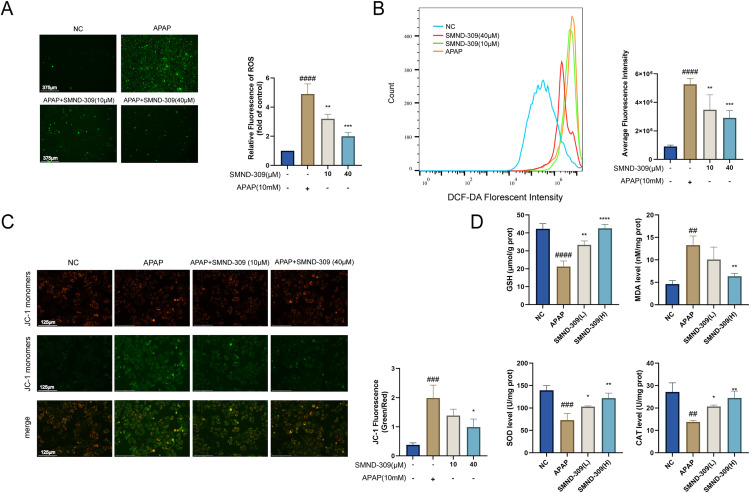
Effect of SMND-309 on APAP-induced oxidative stress and mitochondrial dysfunction *in vitro* and *in vivo.* HepG2 cells were pre-cultured with different doses of SMND-309 (10 and 40 μM) for 24 h, respectively, followed by exposure to 10 mM APAP for 24 h. A. The effect of SMND-309 on ROS levels in HepG2 cells was assessed using a DCFH-DA fluorescent probe. B. The effect of SMND-309 on oxidative stress in HepG2 cells was assessed by flow cytometry after labeling with a DCFH-DA fluorescent probe. C. The effect of SMND-309 on ΔΨm was detected by JC-1 fluorescence probe. The red/green fluorescence ratios were calculated and displayed as bar graphs to reflect the mitochondrial functional status. D. Levels of MDA, GSH, SOD, and CAT in liver tissues of mice from each group were measured. Animal grouping was as described previously. (Statistical plots of the data are expressed as mean ±  standard deviation, with sample size **n** =  3).

To investigate whether SMND-309 can reduce ROS levels, fluorescence microscopy was used in combination with flow cytometry to detect intracellular ROS levels after drug administration. As shown in Fig 3A-B, ROS levels were significantly elevated in the APAP group compared to the NC group, which can be reduced in a concentration-dependent manner by the pretreatment with different concentrations of SMND-309. Further, we investigated the effects of SMND-309 on mitochondrial damage in HepG2 cells. As shown in Fig 3C, normal cells stained with the JC-1 fluorescent probe predominantly emitted red fluorescence, whereas APAP-treated HepG2 cells mainly exhibited green fluorescence. The shift from red to green fluorescence reflects the degree of mitochondrial damage. We found that pretreatment with different concentrations of SMND-309 (10,40 μM) significantly attenuated the APAP-induced increase in green fluorescence intensity, indicating that SMND-309 mitigated the APAP-induced depolarization of mitochondrial membrane potential (ΔΨm). Additionally, we measured the levels of MDA in liver tissues from different experimental groups of mice and found that SMND-309 alleviated the APAP-induced increase in MDA levels (Fig 3D). Moreover, we examined the effects of SMND-309 on the levels of intracellular antioxidant substances. The results showed that SMND-309 significantly increased the levels of SOD, CAT, and GSH in APAP-treated liver tissues, especially at a dose of 60 mg/kg.

### 3.4. Effects of SMND-309 on Keap1-Nrf2 signaling pathway and Nrf2 nuclear translocation in vitro

**Fig 4:**
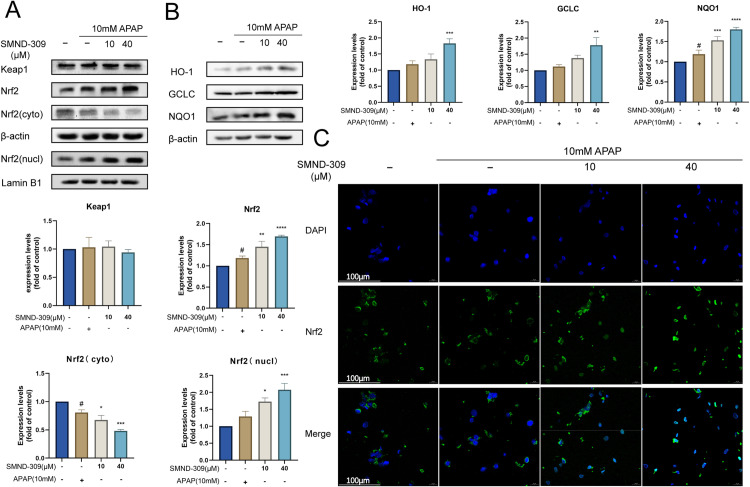
The regulatory effect of SMND-309 on the Keap1-Nrf2 signaling pathway and its effect on the expression of antioxidant proteins in HepG2 cells. HepG2 cells were first pre-cultured with different concentrations of SMND-309 (10 μM and 40 μM) or serum-free medium for 24 h, followed by the co-culture with 10 mM APAP for 24 h. A. The effect of SMND-309 on the expressions of Keap1 and Nrf2 was detected by Western Blotting. B. The regulatory effect of SMND-309 on the expressions of antioxidant proteins (HO-1, GCLC, and NQO1) was analyzed by Western Blotting. C. The effect of SMND-309 on Nrf2 nuclear translocation was assessed by immunofluorescence. (Statistical plots of the data are expressed as mean ±  standard deviation, with sample size n =  3).

To investigate whether SMND-309 affected the Nrf2 signaling pathway, changes in the expression of key proteins of this pathway were detected. As shown in Fig 4A, SMND-309 treatment significantly elevated the total intracellular Nrf2 content and promoted the intranuclear translocation of Nrf2 protein. The assay of Keap1 content was also performed to clarify the specific effects of SMND-309 on specific cascade parts in the Keap1-Nrf2-ARE pathway. However, it is noteworthy that the content of Keap1 protein was not significantly different after SMND-309 treatment compared with normal control or APAP-treated groups. It was also found that the contents of proteins such as HO-1, NQO-1, and GCLC downstream of Nrf2 were also significantly elevated (Fig 4B). Immunofluorescence experiments revealed that the intranuclear translocation of Nrf2 was significantly promoted compared with the model group (Fig 4C).

### 3.5. Effects of SMND-309 on Keap1-Nrf2 signaling pathway and Nrf2 nuclear translocation *in vivo*

**Fig 5:**
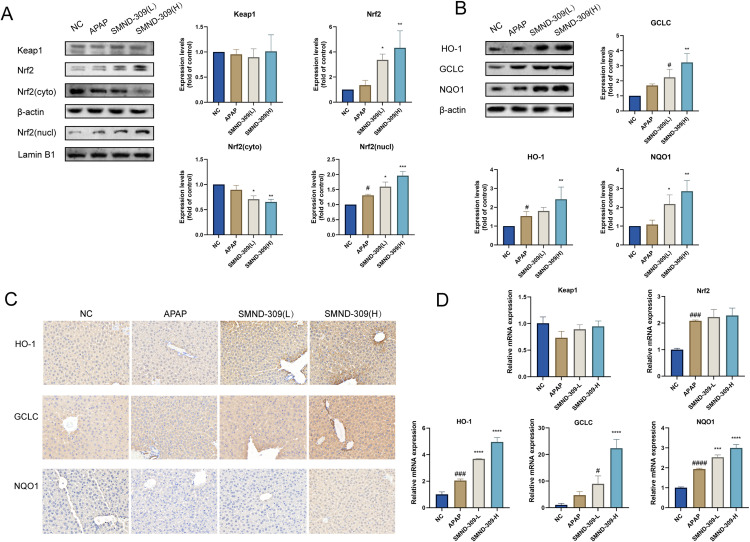
Effect of SMND-309 on Keap1-Nrf2 signaling pathway in liver tissues of the mouse model. 24h after APAP administration, mice were executed to obtain liver tissue. A. The effect of SMND-309 on the expressions of Keap1 and Nrf2 in the nucleus and cytoplasm of cells was detected by Western Blotting. B. The effect of SMND-309 on the expressions of antioxidant proteins (HO-1, GCLC and NQO1) was assessed by Western Blotting. C. The effect of SMND-309 on the content of antioxidant proteins (HO-1, GCLC and NQO1) in liver tissues was evaluated by immunohistochemistry. D. The effect of SMND-309 on the expressions of Keap1 and Nrf2 in cell nucleus and cytoplasm was evaluated by real-time quantitative analysis. D. Effect of SMND-309 on the mRNA levels of Keap1, Nrf2, and antioxidant proteins (HO-1, GCLC, and NQO1) was detected by real-time quantitative PCR. (Statistical plots of the data are expressed as mean ±  standard deviation, with sample size **n** =  3).

To further investigate the improvement mechanism of SMND-309 on AILI *in vivo*, its effect on the Keap1-Nrf2 pathway was studied using BALB/c mice. Pretreatment with SMND-309 promoted not only the expression and nuclear translocation of Nrf2 (Fig 5A), but also the expressions of HO-1, GCLC, and NQO1 proteins in liver tissues (Fig 5B), which is consistent with the results of *in vitro* experiments. As shown in Fig 5C, immunohistochemical experiments further confirmed the induction of HO-1, NQO1, and GCLC proteins by SMND-309 in liver tissues. The expressions of Keap1-Nrf2 pathway-related genes at the transcriptional level were further detected using qPCR. SMND-309 treatment significantly enhanced the transcriptional activities of HO-1, NQO-1, and GCLC (Fig 5D). However, the mRNA content of Nrf2 did not show significant changes even under SMND-309 treatment at a concentration of 40 μM. The above experimental results showed that SMND-309 promoted Nrf2-ARE signaling activation without increasing gene transcription of Nrf2.

### 3.6. Molecular docking and molecular dynamics simulations

**Fig 6:**
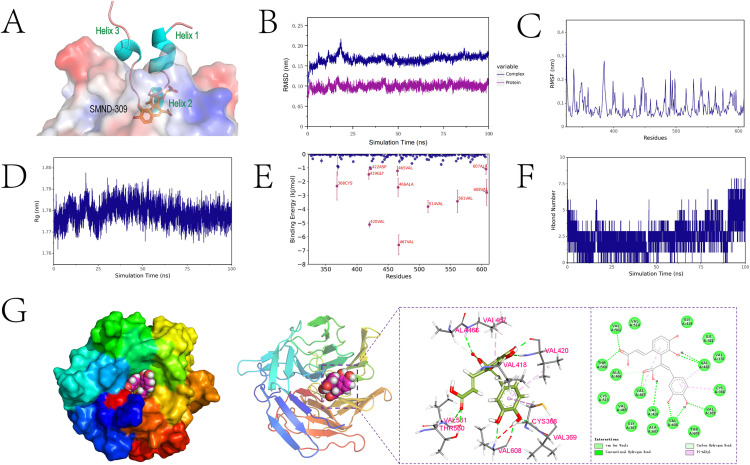
Integrated analysis of molecular docking and molecular dynamics simulation reveals the molecular mechanism of SMND-309-Keap1-DC interaction A. Demonstration of an accurate 3D docking model of SMND-309 and Keap1-DC complex based on the Protein Data Bank (PDB) structure 3WN7. B. Analysis of the RMSD of SMND-309-Keap1-DC complex, protein monomer RMSD during the simulation. C. Assessment of RMSF of the ligand-receptor complex during the simulation. D. Calculation of the Rg of the complex. E. Analysis of the contribution of individual amino acid residues to the binding energy of SMND-309 to Keap1-DC. F. Statistics on the number and dynamics of hydrogen bonds during the simulation. G. MD simulations of the SMND-309-Keap1-DC complexes with detailed 3D and 2D binding modes, including key interactions and binding pocket characteristics.

To explore the interaction of SMND-309 with Keap1, SMND-309 was localized to the Nrf2-binding domain of Keap1 by molecular docking. Fig 6A shows that SMND-309 and DLG motifs in the Keap1 pocket significantly overlap with the Helix-2 region of Nrf2. It was observed by molecular dynamics simulations that the protein-ligand complex remained stable within 100 ns, and the RMSD (Root Mean Square Deviation) data showed that the complex reached a stable plateau after an initial rise and fluctuated between 1.5 Å and 1.8 Å, suggesting the stable ligand binding (Fig 6B). It was found by RMSF (Root Mean Square Fluctuation) analysis that regions with smaller fluctuations were relatively stable in the simulations, which may be key interacting residues (Fig 6C). The overall stability of the complex structure was demonstrated by Rg analysis (Fig 6D).

Further, the difference in bond energies before and after binding of the small molecule-protein complexes was calculated using the MM-PBSA method to obtain ΔE_MMPBSA_ =  -23.178 ±  10.753 kJ/mol, indicating that the binding process releases energy. After the decomposition of ΔE_MMPBSA_, amino acids such as VAL-467 and VAL-420 were found to contribute significantly to the binding energy (Fig 5E), with the number of hydrogen bonds between small molecules and proteins fluctuating between 2-5 (Fig 5F). Finally, the conformation at the end of the simulation was analyzed to find that residues such as VAL-561, THR-560, and VAL-467 formed hydrogen bonds with the small molecule, which are essential for binding stability (Fig 5G).

### 3.7. BLI vindication for SMND-309-Kelch interactions

**Fig 7:**
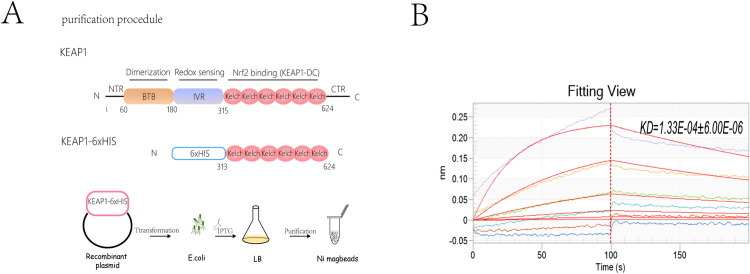
A. Structure and purification process of Keap1 recombinant protein with functional fragments highlighted. B. The binding affinity of SMND-309 to Keap1 was determined using BLI, with curves presenting real-time mapping of sensor assays.

To further validate the interaction between SMND-309 and Keap1, a Kelch recombinant protein containing the 312-624 locus was expressed and purified for subsequent Biolayer Interferometry (BLI) experiments (Fig 7A), with the results showing significant binding between SMND-309 and Keap1 with a dissociation constant KD of 1.33E-04 ±  6.00E-06 (Fig 7B). Combined with molecular dynamics simulation experiments, these results suggest that SMND-309 can bind to the Kelch-binding domain to exert its biological effects.

### 3.8. SMND-309 improved APAP-induced oxidative stress in an Nrf2-dependent manner

**Fig 8:**
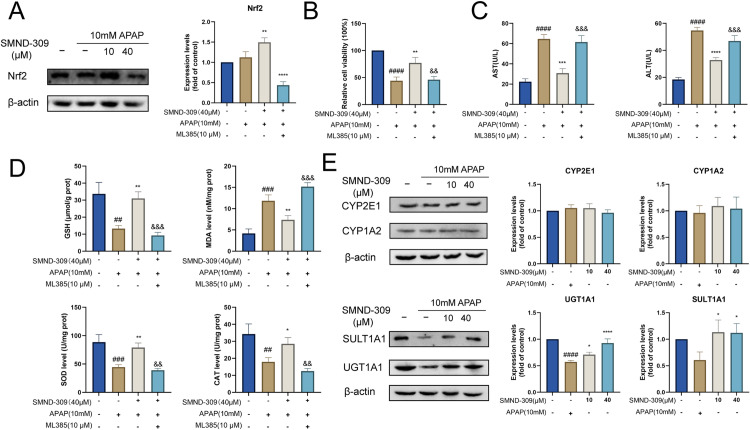
SMND-309 attenuated APAP-induced cell death and oxidative stress in HepG2 cells through an Nrf2-dependent manner. HepG2 cells were first pre-treated with the Nrf2 inhibitor ML385 (10 μM) for 24 h, followed by establishing cellular models as described previously. (A) The effect of SMND-309 on Nrf2 expression was detected by Western Blotting. (B) The effect of SMND-309 on HepG2 cell viability was assessed by MTT assay. (C) Determination of AST and ALT contents in the supernatants of HepG2 cells in each group. (D) Determination of MDA, GSH, T-SOD and CAT in HepG2 cells in each group. E. Effect of SMND-309 on the expressions of I-phase metabolizing enzymes (CYP2E1 and CYP1A2) was analyzed by Western blotting. F. Effect of SMND-309 on the expressions of phase II detoxification enzymes (UGT1A1 and SULT1A1) was analyzed by Western blotting. Differences between adjacent groups are indicated by the symbols. (Statistical plots of the data are expressed as mean ±  standard deviation, with sample size n =  3).

To further validate the critical role of the Nrf2 signaling pathway in the pharmacological effects of SMND-309, cells were treated with ML385 (Nrf2 inhibitor) to block Nrf2-ARE signaling. It was found in the Western Blotting assay that ML385 can effectively reduce Nrf2 expression in HepG2 cells (Fig 8A). It was also found in the study that the survival rate of cells pretreated with SMND-309 was significantly decreased after blocking Nrf2 signaling compared with the experimental group in which the Nrf2 signaling pathway was not blocked (Fig 8B), with AST and ALT levels significantly increased in supernatants (Fig 8C). In addition, SOD, CAT and GSH contents in HepG2 cells were found to be decreased after blocking the Nrf2 pathway, with MDA levels, however, showing an increasing trend (Fig 8D). These results showed the cytoprotective effect and antioxidant activity of SMND-309 for the AILI model significantly decreased after blocking Nrf2 signaling.

### 3.9. Effects of SMND-309 on the detoxification enzymes of APAP

To further investigate the effects of SMND-309 on the key metabolic enzymes of APAP, the effects of SMND-309 on the expressions of CYP450 enzyme family and phase II detoxification enzymes were further explored by Western blotting, with the results showing that the expression levels of CYP2E1 and CYP1A2, which are the key enzymes for converting APAP to its toxic metabolite NAPQI, were not significantly affected in the SMND-309-treated experimental group (Fig 8E). In contrast, the expression levels of phase II detoxification enzymes (UGT1A1 and SULT1A1), which are involved in APAP scavenging, were elevated after SMND-309 pretreatment. This finding suggested that SMND-309 can exert a protective effect against AILI and enhance the levels of detoxification enzymes in the liver without inducing CYP450.

## 4. Discussion

APAP is a commonly used drug for the treatment of pain and fever, which is also one of the most significant contributors to drug-induced liver injury (DILI), with its triggering of AILI posing a major public health challenge [23]. Untreated AILI is likely to progress to acute liver failure (ALF) with the patient survival rate of less than 30% [24], at which time liver transplantation will become a mandatory treatment. Therefore, the search for effective treatments for AILI is the endeavor of many researchers.

In recent years, a variety of natural products have shown therapeutic potential for AILI [17]. Previous studies have shown that SMND-309 can effectively alleviate carbon tetrachloride-induced hepatotoxicity [15], with its therapeutic effect on AILI remaining unclear. It has been shown that oxidative stress plays a key role in APAP-induced hepatotoxicity. NAPQI, a toxic metabolite produced by the metabolism of excess APAP in the liver, irreversibly binds to protein sulfhydryl groups after GSH depletion to form NAPQI-protein adducts (NAPQI-ADs) [25, 26], which is a key link between oxidative stress, mitochondrial dysfunction and cell death [27]. The intracellular GSH content and oxidative stress indexes were detected to investigate the potential therapeutic effects of SMND-309 on AILI, with the results showing that SMND-309 can restore the GSH content and significantly reduce the ROS level and MDA content in APAP-treated HepG2 cells. T-SOD and CAT are important antioxidant components against APAP-induced hepatotoxicity, playing a key role in maintaining the redox balance and defense against oxidative stress in hepatocytes. It was shown in the study that SMND-309 can restore the activity of these antioxidant components in a concentration-dependent manner. The extent of APAP-induced mitochondrial damage can be reflected by changes in ΔΨm [6], which can be restored by SMND-309 to a certain extent to alleviate mitochondrial dysfunction. The study results of Zhou et al. [28] were consistent with our result that water extract from salvia miltiorrhiza can protect hepatocytes from APAP-induced injury by reducing oxidative stress and maintaining mitochondrial metabolic activity. Subsequently, the molecular mechanisms underlying the hepatoprotective effects of SMND-309 were further investigated.

Keap1 acts as a major upstream repressor of the Nrf2 signaling pathway regulating the nucleus translocation of Nrf2 [29]. In the Nrf2-mediated transcriptional regulatory network, GCLC is a key determinant of GSH synthesis rate as the catalytic subunit of γ-glutamylcysteine synthetase (γ-GCS). In addition, HO-1 and NQO1 play an important role in reducing ROS generation as endogenous antioxidants. It has been reported that natural products such as Baicalein [30], Chlorogenic Acid [30] and Puerarin [22] can promote the *in vivo* synthesis of GSH and resist ROS by enhancing the nucleus translocation of Nrf2 and the expressions of downstream key proteins like GCLC to alleviate AILI, with Baicalein and Chlorogenic Acid activating the nuclear translocation of Nrf2 by blocking the direct interaction of Keap1 with Nrf2 and inducing the phosphorylation of ERK1/2. It was shown in the study that SMND-309 significantly enhanced the nucleus translocation of Nrf2 and increased the protein expressions of the antioxidant enzymes HO-1, NQO-1, and GCLC as well as their transcript levels. It was also observed in further experiments that the cytoprotective effect and anti-oxidative stress capacity of SMND-309 were significantly reduced after blocking Nrf2 signaling using ML385. Therefore, it was demonstrated in the study that SMND-309 can promote intracellular antioxidant synthesis in a Keap1-Nrf2-ARE signaling pathway-dependent manner. In addition, the use of APAP alone was observed in combination with previous studies to activate the Nrf2 signaling pathway *in vitro* experiments with a weak effect, which was not observed with the use of H_2_O_2_ as an inducer of oxidative stress [14,22]. Therefore, it was hypothesized that cells may resist oxidative stress injury by spontaneously activating the Nrf2 signaling pathway under oxidative stress conditions. In contrast, H_2_O_2_ may directly cause cell dysfunction and cell death through a non-Nrf2-dependent pathway.

However, it was demonstrated in the study that the Keap1-Nrf2-ARE signaling pathway was activated by SMND-309 without significant changes in the mRNA content of Nrf2, with the Keap1-Nrf2-ARE axis functioning through a unique hinge-lock mechanism [31]. The ETGE (“hinge”) and DLG (“latch”) structural domains in Nrf2 interacted with the Keap1 C-terminal Kelch structural domain to recruit the ubiquitination ligase complex to promote Nrf2 ubiquitination labeling. Currently, biophysical studies have demonstrated that some natural products like Resveratrol [32] and Ginsenoside CK [33] can block the Keap1-Nrf2 interaction and activate the Nrf2 signaling pathway by binding to Keap1 in a non-covalent manner. Molecular docking analysis was performed to further elucidate the molecular mechanism of Keap1-Nrf2 activation by SMND-309. After successfully docking SMND-309 with the Kelch structural domain, SMND-309 was superimposed with the DLG structural domain to show that SMND-309 occupied the binding site of DLG-Helix-2 in the Kelch hydrophobic pocket. Further, the MD results showed that SMND-309 binds stably to Kelch, with strong binding energy and affinity and a small molecule-to-protein ΔE_MMPBSA_ =  -23.178 ±  10.753 kJ/mol, which is the same as other PPI inhibitors. SMND-309 utilizes the natural regulatory mechanism of Keap1 for Nrf2 to reduce Nrf2 ubiquitination and promote its nuclear translocation by competing with Nrf2 for the hydrophobic pocket on the surface of Keap1. Notably, Zhong et al. identified the novel KEAP1-Nrf2 PPI inhibitor ZT0633 [34] by FA screening assay and X-ray crystal diffraction. Structurally, ZT0633 is a variant of the monomer contained in SMND-309, which is characterized by the lack of a hydroxyl group on the benzene ring. Therefore, SMND-309 was supposed to have similar biological activity. Subsequently, the actual interaction of SMND-309 with Keap1 was successfully detected by BLI experiments, further providing evidence for the potential interaction of SMND-309 with Keap1. It was also demonstrated that SMND-309 can directly interact with the Nrf2 binding pocket of Keap1 through the PPI mechanism of action to inhibit Keap1-mediated Nrf2 ubiquitination and activate the Nrf2-ARE signaling pathway, which reveals the reason why SMND-309 promotes nuclear translocation by activating Nrf2 only at the protein level and not dependent on its elevated mRNA level.

Currently, NAC is the only drug approved by the FDA for the treatment of AILI. However, NAC has many disadvantages, like leading to adverse effects like rash and asthma, with its clinical use limited to a narrow therapeutic window in the early stages of the disease [7]. In addition, there is growing evidence that some natural products may be more effective than NAC in the treatment of AILI with lower adverse effects [17,35]. Our study results demonstrated that SMND-309 can increase the levels of detoxification enzymes in the APAP metabolic pathway and exert hepatoprotective effects without inducing the production of the CYP450 enzyme system, preventing further induction of NAPQI production. This suggests that the clinical application of natural products such as SMND-309 or the use of NAC-natural product combinations may enhance the clinical efficacy and improve the disease prognosis in the treatment of AILI.

## 5. _Conclusion_

**Fig 9:**
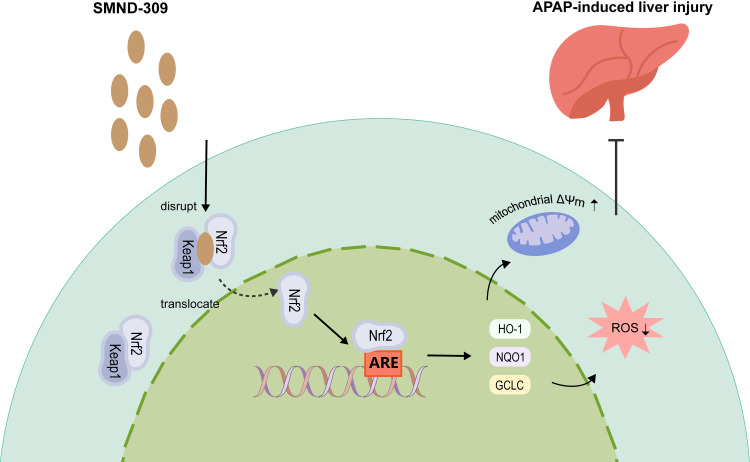
Pharmacological mechanism of SMND-309.

In conclusion, our study demonstrated the significant hepatoprotective effect of SMND-309 against AILI, with SMND-309 significantly reducing serum AST, ALT and LDH levels and attenuating pathological changes in liver tissues in mice. In addition, SMND-309 activated the Nrf2-ARE signaling pathway by inhibiting the interaction between Keap1 and Nrf2, restoring antioxidant enzyme activities, and attenuating ROS accumulation and mitochondrial damage (Fig 9). These findings provide sufficient evidence for the hepatoprotective properties of SMND-309 and provide new ideas for clinical prevention and treatment of AILI.

## Supporting information

S1 File Raw images. (PDF)

S2 File Raw Data1. (RAR)

S3 File Raw Data2. (RAR)

S4 File Raw Data 3. (RAR)
