# Supplementary material for: SMND-309 activates Nrf2 signaling to alleviate acetaminophen-induced hepatotoxicity and oxidative stress
Source: PLoS One. 2025 Mar 31;20(3):e0310879. doi: 10.1371/journal.pone.0310879 (PMC11957308; doi:10.1371/journal.pone.0310879)

Note: Some bands are prefixed with the same letters or numbers, indicating that they originate from multiple incubations of the same membrane, hence different target proteins may display the same internal reference. We treated the PVDF membrane with an antibody stripper to thoroughly remove the antibodies.

fig.4 A

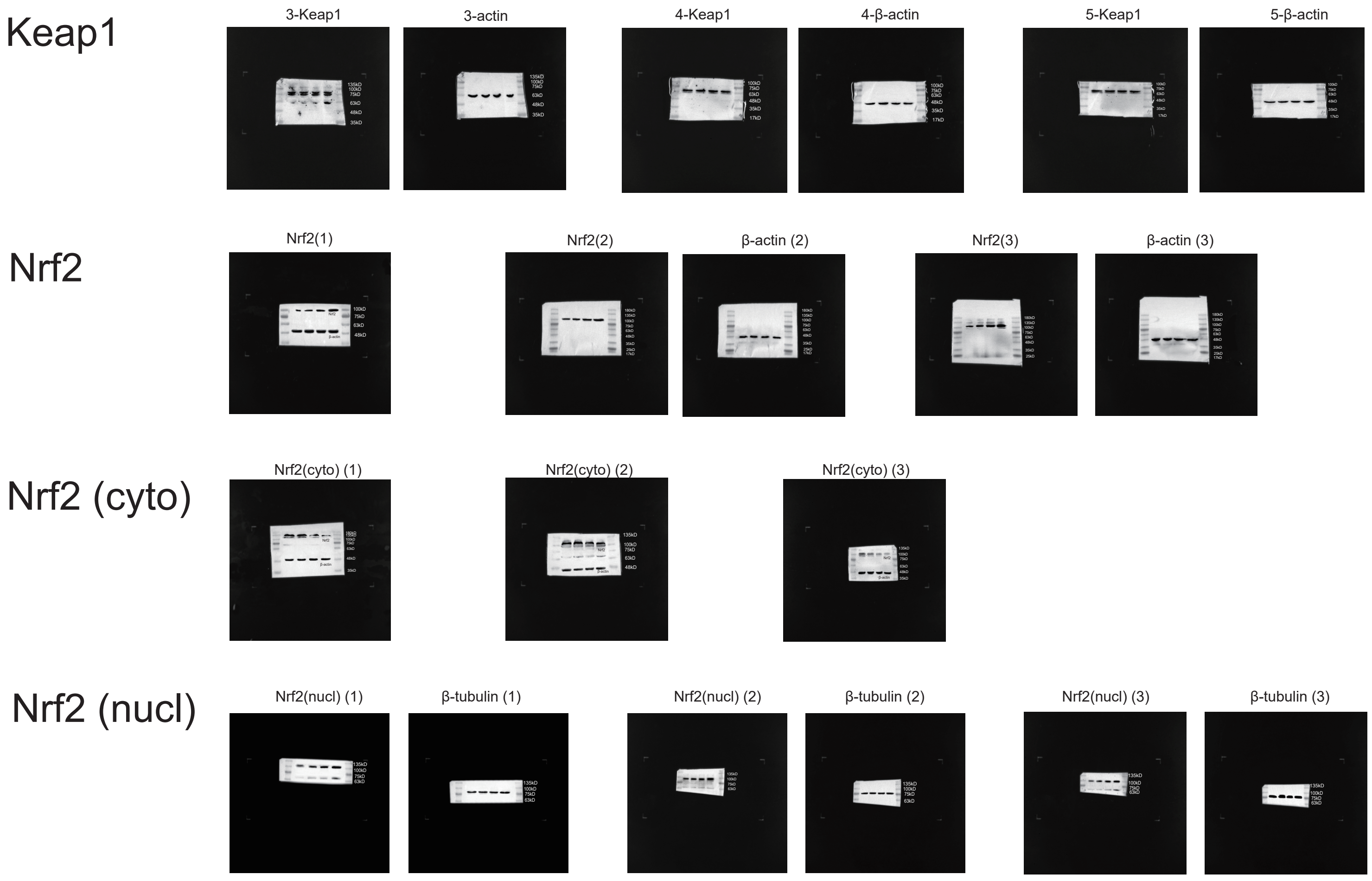

fig.4 B

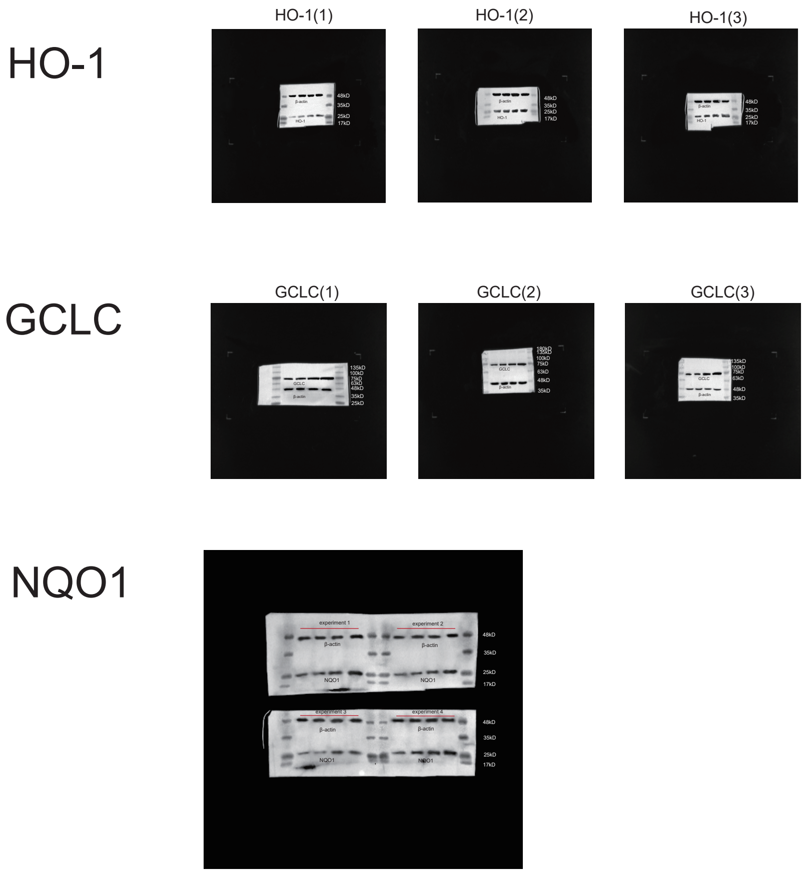

fig.5 A

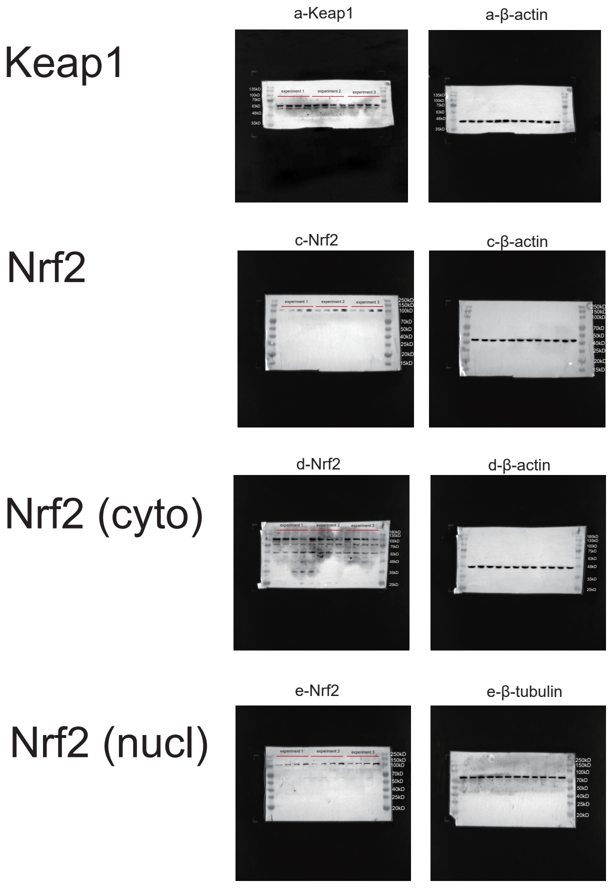

fig.5 B

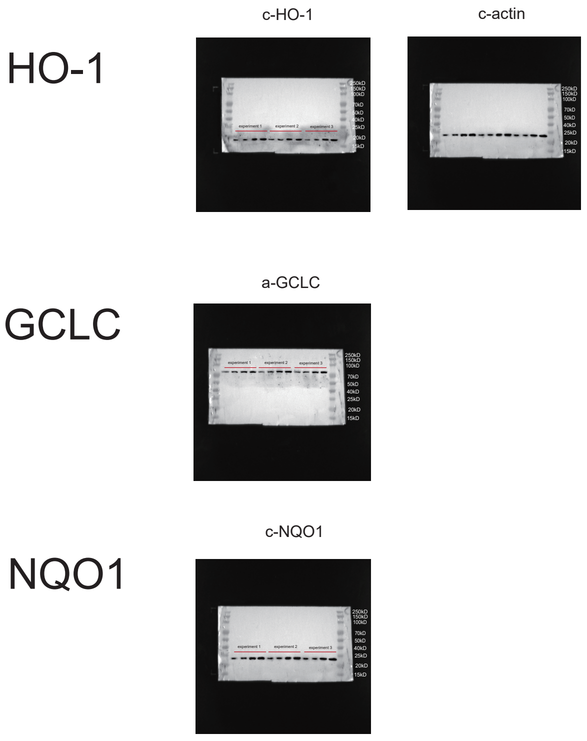

fig.8 A

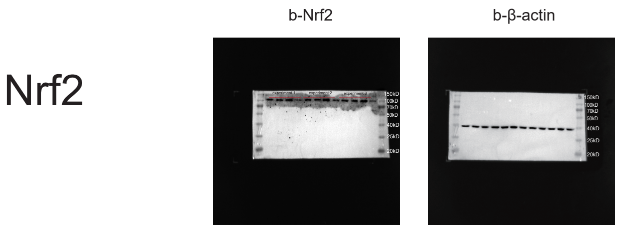

fig.8 E

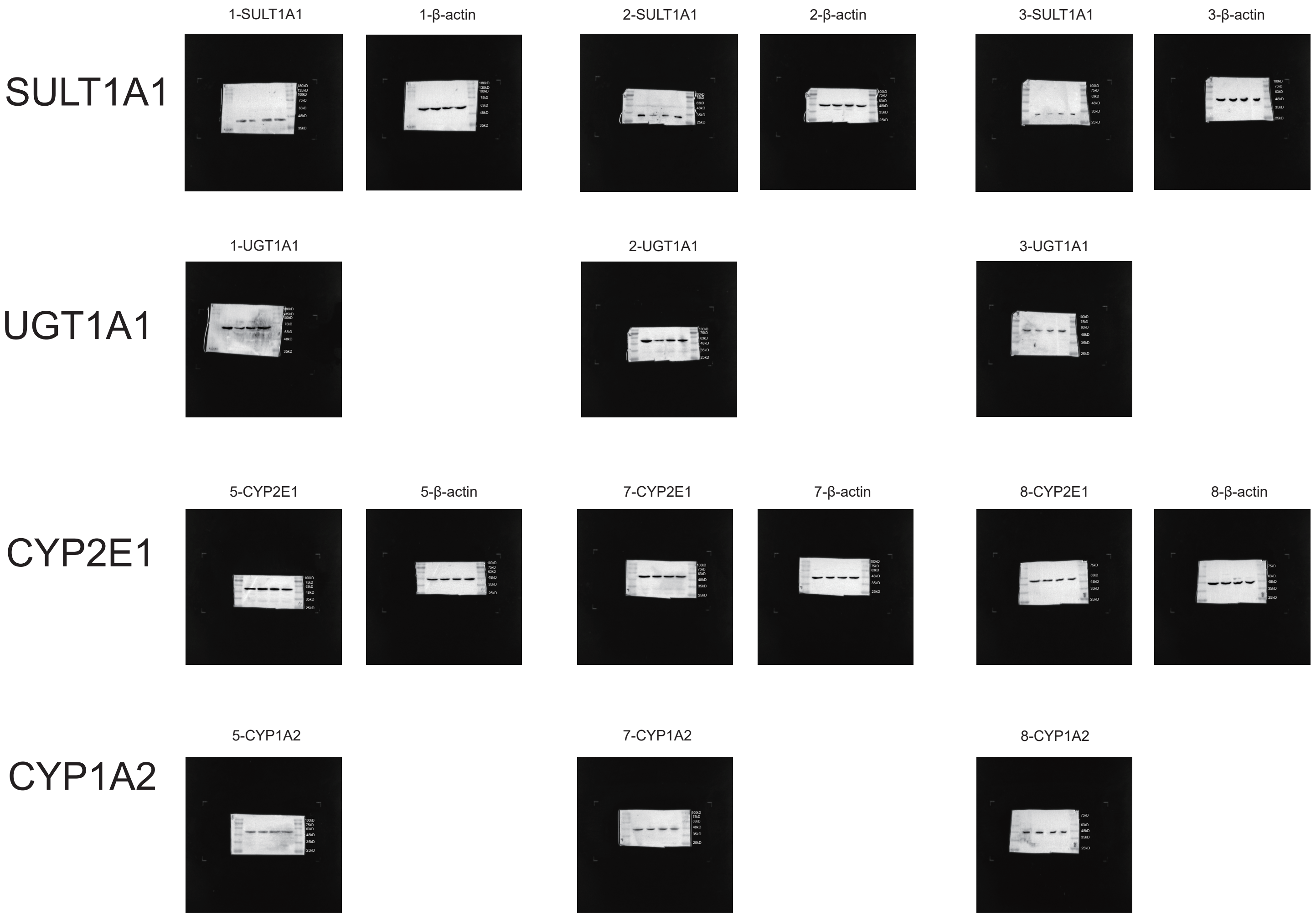

Supplement: S1 File — (PDF) [file pone.0310879.s001.pdf]
